# Doctor-patient communication: Patient perception

**DOI:** 10.4103/0019-5545.37309

**Published:** 2007

**Authors:** G. Swaminath

**Affiliations:** Department of Psychiatry, Dr. B. R. Ambedkar Medical College, Kadugondanahalli, Bangalore - 560045, India

## NO LONGER PATIENT

While chatting with a popular and meticulous general practitioner about his referrals to psychiatrists, he remarked that he found his patients “less than satisfied” with psychiatric consultations. This set me thinking of an experience I had had the previous day.

That evening, I had two demanding patients - Ms. A and Ms. B - in successive sessions, of whom the second, Ms. B, on arrival informed me that the first patient, Ms. A, was complaining to her husband while leaving my consulting room of her dissatisfaction with the consultation. Later after I had “finished” with Ms. B, she remarked, “Now I too am dissatisfied” and promptly stomped out.

Reflecting on what happened, I realized that I had seen two difficult categories of patients. Ms. A had been insisting that she did have a major physical disorder which had not been recognized in her previous consultations, was reluctant to consult a psychiatrist, denied any psychological stressors and complained that she did not believe that her illness could be due to psychological factors and that despite this was advised otherwise and prescribed “sedatives.” I too had felt unsatisfied with the consultation as she left on a sour note. Ms. B, on the other hand, did not believe she had a problem, insisted that her suffering was due to issues with her husband's family and was reluctant to take drugs for someone else's “mistake.” She was unhappy at being told that her fears and suspicions about her husband's family could be part of her belief system and that she would feel less animosity and more calm with drugs. She left suspecting me of conspiring with her husband's family against her. Both Ms. A and Ms. B have not since come back for consultation.

## PHYSICIAN-PATIENT COMMUNICATION

At its worst, patient dissatisfaction could be fatal to the treating physician. The case of 50-year-old Dr. Vasant Waman Jaykar, a cardiologist of Mumbai, who was shot dead in January 2001 by a deceased patient's dissatisfied relative, brings home the point.[1] Thankfully, dissatisfaction very rarely takes this extreme form; nor does it cause patients to lean towards the other extreme, i.e., the Gandhian approach of showing the other cheek. The reaction is generally muted like in the case of A and B, who stopped at voicing their rejection of the physician. Such rejection may at times be more passive. All these responses may seriously harm the physician.

Research has shown that the way patients perceive their connection with their physician significantly influences their sense of satisfaction and level of concern about their health.[2] In the fast-paced managed-care environment, relationship-building conversations can get lost in the pressure to perform. The demands of keeping abreast of the latest medical-treatment approaches can overshadow the need to practice and improve communication skills. A good effective, empathic physician-patient communication leads to improved patient compliance, better clinical outcomes and reduction in “doctor-shopping” and malpractice litigations.[3]

Neuwirth[3] notes that good communication is good business practice. He further suggests that contrary to general assumptions, communication skills are not strictly inborn assets or talents but rather skills that can be learned and developed – much like those needed to learn to play a musical instrument. The musical instrument metaphor is particularly apt, as playing the instrument well requires continuous practice and improvement, regardless of the level of talent. The need to address physician-patient communication lies in the necessity to continuously improve and hone one's ability to communicate in ways that build and sustain positive patient relationships. The importance of patient's perceptions, physician empathy and communication style can never be overemphasized.[4]

## PATIENT PERCEPTIONS MATTER

Many of our patients complain that the duration of consultation was inadequate and that the consultation was hurried. Physicians commonly redirect and focus clinical interviews on issues they consider important, before giving patients even the opportunity to complete their statement of concerns. Kim Marvel and others[5] suggested that patients, asked to describe their concerns by a physician, were most often redirected after the first expressed concern and after a mean time of only 18–23 seconds. The frequency with which experienced physicians solicited the patient's complete agenda is quite low (28%). This resulted in missed opportunities to gather potentially important patient data. Once the discussion became focused on a specific concern, the likelihood of returning to complete the agenda was very low (8%). However, going back to the agenda by an open-ended solicitation by asking “Anything else?” repeatedly until a complete agenda has been identified appears to take six seconds longer than interviews in which the patient's agenda is interrupted.

Fossum and Arborelius[6] identified components of patient-centered consultations which resulted in patient satisfaction. The main characteristics were flexibility, faster pace and frequent movement back and forth between discussion topics and communication tasks – for example, a fluid movement between discussing the etiology of the problem, patient concerns and patient expectations. In contrast, a steady, slow, sequential movement through the topics was associated with patient dissatisfaction, most likely because this style entailed little or no “shared understanding” or “involving patient” communication.

Effective communication can be difficult under the best of circumstances. When the patient is emotionally distressed, extra effort is required to ensure that the patient accurately perceives what is communicated. Attending to the patient's emotional state improves the patient's perception of the communication exchange.[4]

Thus, it seems that waiting for patients to air their concerns completely before redirecting them and later focusing on each concern seems ideal. However, to focus on each concern as and when the patient airs it and then continue with an open-ended solicitation until the complete agenda of the patient is met could well be another acceptable option. The latter option takes hardly six seconds more than when the interview is interrupted. Finally, a fluid movement between topics rather than a sequential movement through the topics led to better patient satisfaction. Greater patient satisfaction was associated with greater interview length, increases in the proportional time spent by the physician in presenting information and discussing prevention and shorter chart review times.[7]

## EMPATHY AND PATIENT SATISFACTION: THE VALUE OF THE RELATIONSHIP

In psychotherapy, as well as in the general practice of medicine, the empathic relationship is primary. Without a sense of connection and mutual understanding, physician-patient communication becomes an exchange of medical information divorced from the context and complexities of the patient's life. Current research highlights the need to recognize the importance of attending to the patients' emotion-laden comments and to improve empathic skills.

Patient satisfaction and treatment compliance were shown by Kim and colleagues[8] to relate directly to a physician's empathic behavior. In their study, perception of physicians' “affective empathy” and “sense of partnership” had the strongest impact on patient satisfaction and compliance. By contrast, “cognitive empathy” and “information sharing” had little effect on patient satisfaction and a negative effect on compliance. Kim and colleagues concluded that improving empathic communication skills might be the most effective approach to improving patient satisfaction and treatment compliance.

The patients in the study by Zachariae and colleagues,[9] rated the importance of physicians' ability to listen and communicate on par with their ability to respond to emotional needs. They showed that patient distress was negatively correlated with physician attentiveness and empathy, and patient self-efficacy was positively correlated with attentiveness and empathy.

Parchman and colleagues' findings[10] support what is intuitively understood about physician-patient relationships – that the length of relationship increases trust and communication effectiveness.

It could therefore be concluded that affective empathy and a sense of partnership as against merely information sharing lead to better patient compliance and self-efficacy.

## COMMUNICATION STYLE AND APPROACH

There is a belief among doctors that being proficient and delivering specialized service is more valuable than human values and doctor-patient relationship. This leads to physicians adopting a dispassionate approach when communicating with patients.

The age-old belief that how you say something matters more than what you say was borne out by few studies examining physician communication style and patient response. Heisler and colleagues[11] found that physicians' ability to provide illness-related and treatment-related information in such a way that patients fully understood the importance of self-management was predictive of good self-management practices.

Engaging in social talk contributed to a perception of being understood, according to a study by Takayama and Yamazaki.[12] Here, patients perceived open-ended questions and a “partnership-building approach” as contributing to their sense of mutual participation. The physicians' provision of medical information and counseling increased the patient's perception of “self-participation.” There was a strong correlation between their sense of “successful communication” and their sense of participation and the “physician's collaborativeness.” The authors suggested that sensitivity to the patient's emotional state could help to improve patients' perception of successful communication.

Ambady and colleagues[13] examined the tone of voice from short clips taken at the beginning and end of surgeons' consultations. Of the surgeons who participated in the study, half had been involved in malpractice claims and half had not. Using analysis of tone of voice only, analyzers, who were blind to the physicians' claims history, were able to identify surgeons with a history of malpractice claims. Analyzers' higher ratings of “dominance” and lower ratings of “concern/anxiety” were predictive of malpractice history.

This suggests that a communication style sensitive to patient's emotional state, as well as one which improves bonding with patient, improves patient outcome and reduces patient dissatisfaction.

## DISADVANTAGES FACED BY PSYCHIATRISTS

Patients who consult psychiatrists are noncooperative as many lack insight into their illness. Many land up with multiple consultations and investigations rather than seeing a psychiatrist. This interferes with rapport-building and establishment of an empathetic relationship in some cases. It has also been shown that patients' illness perceptions before the consultation, in particular, the uncertainty about what was wrong with them and the symptoms making them feel helpless, depressed, etc., predicted patient dissatisfaction. Poor mental health is known to be associated with dissatisfaction. Hence unlike the doctor-patient relationship in the medical setting, the psychiatrist-client relationship seems to start with an inherent disadvantage. Both patient and psychiatrist factors contribute to this difficult relationship. They are:

### Contributions of the patient

- Stigma
- Denial of psychiatric causation of illness
- Discomfort in revealing inner/hidden painful material
- Lack of trust in others
- Unfamiliarity with psychiatric consultation
- Unrealistic expectations about the consultation

### Contributions of the psychiatrist

- Lack of interest in patient with no fascinating problems
- Irritation with patient for
  - verbosity
  - lack of clarity
  - lack of consistency
- disappointment anger at
  - failure of treatment
  - poor compliance

Psychiatrists need to be aware of, and be sensitive to, the contributions of these factors to poor relationship.

Psychiatrists develop skills that often help people to cope with their mental-health problems, enabling them to make progress towards a solution after other help has failed. People with mental illness are extremely unhappy and difficult to reach, may feel cut off from the rest of the world and find it almost impossible to have trust or confidence in anyone. Their psychiatrist can be the one person who can make a difference and can give hope at the most despairing times. While cures are often difficult to effect, psychiatrists can make an enormous contribution to improving the quality of life of their patients, reducing their symptoms and distress and making an impact on their social conditions.[14]

Thus it is an efficient psychiatrist-patient relationship which serves as a foundation for improved patient satisfaction and compliance.

## REDRESS FOR THE DISSATISFIED

In a panel discussion on medical ethics in Mumbai some time back, discussion veered towards the issue of prominent reports in the newspapers of assaults on medical practitioners by goons in the city at the instigation of aggrieved patients. A well-informed gentleman in the audience asked a question: “A relative has been mistreated by a doctor. What avenues does he have to seek redress?” A member of the panel suggested the State Medical Council and, failing that, the Medical Council of India. “What are the chances of the patient obtaining a fair and prompt hearing? Will these august bodies take immediate action against the erring doctor? How many doctors have been tried by these august bodies; and of these, how many were found guilty in the past decade?” “Alas!” confessed the panelist, “Both these bodies are inefficient and ineffective. To the best of our knowledge few doctors have been tried, though several complaints have been brought before these agencies.” “Is there no other avenue for my relation?” asked the gentleman. “Well, there are the courts of law,” started the panelist. “I'm afraid that's a nonstarter. I should know,” said the gentleman and added, “The courts are clogged and no decision can be expected for decades.” As the panelist shrugged, the gentleman remarked, “In that case, perhaps the underworld has it right. It might be best to bump off the offending doctor.” He was speaking only partly in jest.[1]
